# Colloidal Synthesis and Thermoelectric Properties of CuFeSe_2_ Nanocrystals

**DOI:** 10.3390/nano8010008

**Published:** 2017-12-26

**Authors:** Bing-Qian Zhang, Yu Liu, Yong Zuo, Jing-Shuai Chen, Ji-Ming Song, He-Lin Niu, Chang-Jie Mao

**Affiliations:** 1 The Key Laboratory of Environment Friendly Polymer Materials of Anhui Province, School of Chemistry & Chemical Engineering, Anhui University, Hefei 230601, China; bingqianzhangzbq@163.com (B.-Q.Z.); liuyu92525@gmail.com (Y.L.); mzone2010@hotmail.com (Y.Z.); cjshuai@126.com (J.-S.C.); niuhelin@ahu.edu.cn (H.-L.N.); maochangjie@sina.com (C.-J.M.); 2 Catalonia Institute for Energy Research-IREC, SantAdrià de Besòs, 08930 Barcelona, Spain

**Keywords:** CuFeSe_2_, nanocrystals, colloidal synthesis, ligand removal, thermoelectric property

## Abstract

Copper-based chalcogenides that contain abundant, low-cost and environmentally-friendly elements, are excellent materials for numerous energy conversion applications, such as photocatalysis, photovoltaics, photoelectricity and thermoelectrics (TE). Here, we present a high-yield and upscalable colloidal synthesis route for the production of monodisperse ternary I-III-VI_2_ chalcogenides nanocrystals (NCs), particularly stannite CuFeSe_2_, with uniform shape and narrow size distributions by using selenium powder as the anion precursor and CuCl_2_·2H_2_O and FeCl_3_ as the cationic precursors. The composition, the state of valence, size and morphology of the CuFeSe_2_ materials were examined by X-ray diffraction (XRD), X-ray photoelectron spectroscopy (XPS), scanning electron microscope (SEM), transmission electron microscope (TEM) and high resolution transmission electron microscope (HRTEM), respectively. Furthermore, the TE properties characterization of these dense nanomaterials compacted from monodisperse CuFeSe_2_ NCs by hot press at 623 K were preliminarily studied after ligand removal by means of hydrazine and hexane solution. The TE performances of the sintered CuFeSe_2_ pellets were characterized in the temperature range from room temperature to 653 K. Finally, the dimensionless TE figure of merit (*ZT*) of this Earth-abundant and intrinsic *p*-type CuFeSe_2_ NCs is significantly increased to 0.22 at 653 K in this work, which is demonstrated to show a promising TE materialand makes it a possible *p*-type candidate for medium-temperature TE applications.

## 1. Introduction

During the past few decades, significant increases in the efficiency of thermoelectric (TE) materials have attracted widespread research interest in the development of potential applications for waste heat-to-electricity conversion, cooling and thermal sensing [1,2,3,4,5]. An efficient TE material must exhibit a high TE figure of merit, *ZT*, at the temperature of operation, *T*. The dimensionless TE figure of merit is defined as: *ZT* = *σS*^2^*T/κ*_e_ *+ κ*_L_, where *σ*, *S*, *T*, *κ*_e_ and *κ*_L_ are the electrical conductivity, the Seebeck coefficient, the absolute temperature and the electronic and lattice thermal conductivity, respectively [6,7]. There is no known limitation to *ZT* and, thus, except for the Carnot limit, to the maximum energy conversion efficiency of TE devices. Therefore, TE materials struggle to simultaneously display high electrical conductivities, low thermal conductivities and large Seebeck coefficients, since these three parameters are tightly interrelated. 

Recent progress in the field is related to one efficient way to increase *ZT* by the control of the material’s structure from the mesoscale to the nanoscale in order to scatter phonons in a wide range of wavelengths, resulting in a low thermal conductivity without compromising the electronic properties. The nanostructuration had shown outstanding results or embedding nanoscale precipitates [8], and at the same time, enhancements in their power factors have also been achieved [6], which include introducing a resonance level in the valence band (for example, in Tl-PbTe [9]) or synergistic nanostructuring [10]. Solution processed nanoparticles have emerged as an important opportunity to control the composition and morphology of the nanostructured TE materials at the nano- and meso-scale, and the rational engineering of multicomponent and doped nanomaterials and nanocomposites is possible due to the great control and versatility that the solution processed nanoparticles provide to the construction of highly efficient TE materials [11]. Nevertheless, this is one of the major challenges that has to be solved for colloidal nanomaterials. It needs to be considered how to choose the proper inorganic salts or solvents with the effective process for the ligands’ displacement or organic removal, and it is critical to optimize the transport properties for the final bulk nanostructured material.

However, taking into account the materials’ side, the TE figure of merit at the relevant temperature range needs to be increased using materials that do not incorporate highly toxic or scarce elements such as Pb and Te. In this contribution, copper-based chalcogenide multinary semiconductors, containing abundant, low-cost and environmentally-friendly elements have recently emerged as some of the best performing *p*-type TE materials [12], such as Cu_2_CdSnSe_4_ [13,14], Cu_2_ZnGeSe_4_ [15], Cu_3_SbSe_4_ [16,17,18], Cu_2_SnSe_3_ [19,20,21], etc. One potentially attractive, yet not well-studied, copper-based material is CuFeSe_2_, which represents a well-known class of I-III-VI_2_ group ternary chalcogenides. Among the I-III-VI_2_ group, such as CuInS_2_ [22,23,24], CuInSe_2_ [24,25,26], CuInTe_2_ [27] and CuFeS_2_ [24,28,29,30], materials have attracted extensive attention due to their high absorption coefficient, high conversion efficiency, low toxicity and other physical properties and unique chemical properties and have been explored for the fabrication of photovoltaic solar cells in very recent years. However, among these, less attention been paid to the CuFeSe_2_ material, as eskebornite with a narrow band gap of 0.16 eV belongs to the tetragonal structure type [31] and the crystal structure of the CuFeSe_2_ has the space group P$\overset{-}{4}$2c with cell parameters *a* = 5.521 Å and *c* = 11.04 Å [32].

There are only several reports on the solid-state strategy or surface modified for the synthesis of bulk structures of CuFeSe_2_ materials and the study of the TE properties of compacted dense materials [33] or nanostructured bulk CuFeSe_2_ thin films [34]. The solid-state strategy requires either complex and very high temperature or a long reaction time, and thin films have poorer conductivity due to much lower density. However, up to now, there have been only a few reports on a solution-based synthesis of nanostructured CuFeSe_2_ [35,36,37]. For example, Hsu and his coworkers synthesized cuboid CuFeSe_2_ nanocrystals (NCs) in the presence of solvent octadecylamine without applications [36]. Very recently, Yang and his coworkers reported synthesis of quasi-cubic-shaped CuFeSe_2_ NCs with the magnetic and photoelectric properties by using the reaction of metallic acetylacetonates with diphenyl diselenide (Ph_2_Se_2_) in oleylamine (OLA) with addition of oleic acid (OA) [37]. It is noteworthy that the precursor of Ph_2_Se_2_ is very expensive and toxic, so limiting its scale up, which makes it impossible for TE or other applications for which samples are needed on the order of grams. It is, therefore, not only essential to find a facile way for the large-scale synthesis of high-quality monodispersed and uniform CuFeSe_2_ NCs from low cost chemicals, but also crucial to produce nanostructured materials with a proper *ZT* value. Herein, we present a solution-based scalable synthesis approach to produce several grams of ~6 nm monodisperse CuFeSe_2_ NCs with a uniform shape and narrow size distributions for the production of efficient and environmentally-friendly TE nanomaterials. Furthermore, the composition, the state of valence and the morphology of the CuFeSe_2_ NCs were examined by corresponding test methods. Finally, after organic ligands’ removal, we demonstrate their bottom-up processing into bulk nanostructured materials with high relative density, and the dimensionless figure-of-merit *ZT*_max_ for this pristine CuFeSe_2_ reaches a peak value of 0.22 at 653 K in this work.

## 2. Materials and Methods

### 2.1. Chemicals 

Copper (II) chloride dihydrate (CuCl_2_·2H_2_O, 99.99%), ferric chloride (FeCl_3_, AR), selenium powder (Se, 99.99%), dodecanethiol (DDT, technical grade 98%), oleylamine (OLA, technical grade 70%) and oleic acid (OA, technical grade 99%) were ordered from Aladdin Reagent Co., Ltd. (Shanghai, China). Anhydrous ethanol (CH_3_CH_2_OH, 99%), chloroform (CHCl_3_, AR), hexane (C_6_H_14_, 97%) and hydrazine hydrate (N_2_H_4_·H_2_O, 85%, wt %) were purchased from Sinopharm Group Chemical Reagent Ltd. (Suzhou, China). All chemicals were used without further purification. All the syntheses were carried out using standard airless techniques: a vacuum/dry argon gas Schlenk line was used for the synthesis.

### 2.2. Synthesis of Selenium Precursor Solution 

Selenium powder (1.5792 g, 20 mmol) was dissolved in 20 mL OLA and 20 mL DDT at room temperature, cycled between vacuum and argon to remove the oxygen in the flask, and then stirred under argon atmosphere until the Se powder was completely dissolved.

### 2.3. Synthesis of CuFeSe_2_ Nanocrystals

In a typical synthesis, 10 mmol of CuCl_2_·2H_2_O, 10 mmol of FeCl_3_, 90 mL of DDT and 60 mL of OA were mixed in a 500-mL three-neck flask under magnetic stirring at room temperature with a big heating mantle. The solution was kept at 130 °C under vacuum for 30 min and then heated to 180 °C with argon. Forty milliliters of selenium precursor solution were quickly injected into the reaction under magnetic stirring, and the color of the solution changed immediately from brown to dark, indicating that the nucleation and subsequent growth of CuFeSe_2_ NCs occurred. After injection, the temperature of the reaction mixture dropped to ~160 °C, and it was allowed to recover to the pre-injection temperature. The overall reaction time after recovering to 180 °C was 30 min, and then, the sample was rapidly cooled to room temperature through a water bath. The resultant dark product was thoroughly washed at 6000 rpm for 5 min by multiple precipitation/redispersion steps using chloroform as the solvent and ethanol as a non-solvent. The product was then dried under vacuum and collected for characterization.

### 2.4. Structure and Characterization

The crystal phase was characterized by the AXS D8 ADVANCE X-ray diffractometer (XRD) (Karlsruhe, Germany). X-ray photoelectron spectroscopy (XPS) was used for constant analysis on a VG ESCA θ probe spectrometer (East Grinstead, UK). The size and initial morphology of the product were measured using a ZEISS LIBRA 120 Transmission Electron Microscope (TEM) (Oberkochen, Germany), operating at 120 KV. High resolution TEM (HRTEM) micrographs were obtained using a Tecnai F20 field-emission gun microscope (Hillsboro, OR, USA) with a 0.19 nm point-to-point resolution at 200 kV with an embedded Gatan QUANTUM image filter for electron energy loss spectroscopy (EELS) analyses. The Zeiss Auriga Scanning Electron Microscope (SEM) (Oberkochen, Germany) was used to observe the particle size and morphology at 5.0 kV, and the nanomaterials were analyzed by an Oxford energy dispersive X-ray spectrometer (EDX) attached to Zeiss Auriga (Oberkochen, Germany) SEM at 20.0 kV. The organic molecules on the product surface were measured by Alpha Bruker Fourier Transform Infrared Spectroscopy (FTIR) (Ettlingen, Germany). Thermal gravimetric analyses (TGA) were done using Perkin-Elmer TGA 4000 equipment (Waltham, MA, USA). The dried CuFeSe_2_NCs and pellet pieces were heated up to 600 °C under a nitrogen flow and a heating ramp of 5 °C/min.

### 2.5. Ligand Removal and Bulk Nanomaterial Fabrication

The 3.0 g synthesized CuFeSe_2_ NCs were added to a mixed solution of hydrazine hydrate and hexane with a 1:2 volume ratio to remove the organic long-chain molecules from solvents by the principle of ligand replacement, then stirred continuously for 4–6 h until the NCs easily entered into the hydrazine hydrate phase; next, the hexane phase, which had adsorbed the organic residue, was discarded. New hexane was added into the above hydrazine hydrate phase, and the NCs were washed again. This operation was carried out three times until the supernatant hexane organic phase was clearly transparent, indicating that the organic residue on the NCs’ surface had been completely removed. The clean NCs without organic residue were obtained after centrifuging the excess hydrazine hydrate phase by chloroform after a few minutes of mixing using vortexing and vacuum-dried for hot-pressing. The powders were loaded into a graphite die and compacted into pellets (*Ø*10 mm × 1.5 mm) in an Ar atmosphere using a custom-made hot press for 30 min at a temperature of 623 K under a pressure of 60 MPa. In this system, the heat is provided by an induction coil operated in the RF range applied directly to a graphite die acting as a susceptor, at a temperature 20 °C/s. The density of the pressed pellets was always higher than 90% of the theoretical value.

### 2.6. Thermoelectric Property Measurements

The Seebeck coefficient was measured by using a static direct current (DC) method. Electrical resistivity data were obtained by a standard four-probe method. Both the Seebeck coefficient and the electrical resistivity were measured simultaneously in an LSR-3 LINSEIS system (LINSEIS GmbH, Vielitzerstr, Selb, Germany) in the range between room temperature and 653 K, under helium atmosphere. An XFA 600 Xenon Flash Apparatus (LINSEIS GmbH, Vielitzerstr, Selb, Germany) was used to determine the thermal diffusivities of the pellets. The thermal conductivity was calculated by *κ* = *λC*_p_*ρ*, where *λ* is the thermal diffusivity, *C*_p_ is the heat capacity and *ρ* is the mass density of the specimen. The constant pressure heat capacity (*C*_p_) was estimated from empirical formulas by the Dulong-Petit limit (3R law), and the density (*ρ*) value used here was calculated using Archimedes’ method.

## 3. Results and Discussion

**Figure 1:**
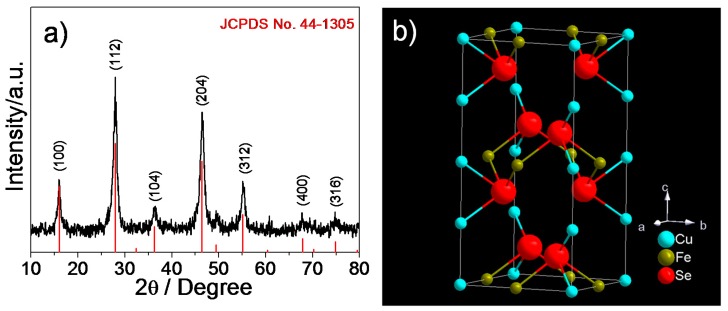
(**a**) X-ray diffraction (XRD) pattern for the obtained samples; the red vertical is the standard literature data; (**b**) unit cell of tetragonal CuFeSe_2_.

From the XRD pattern of the obtained samples (Figure 1a), all observed diffraction peaks are consistent with the standard JCPDS Card No. 44-1305 [38], without any impurity peaks. Several major peaks are shown at 15.99°, 27.9°, 36.31°, 46.43° and 55.08°, respectively corresponding to the (100), (112), (104), (204) and (312) crystal plane of CuFeSe_2_ NCs with a tetragonal structure. Figure 1b displays the typical unit cell structure of the product.

**Figure 2:**
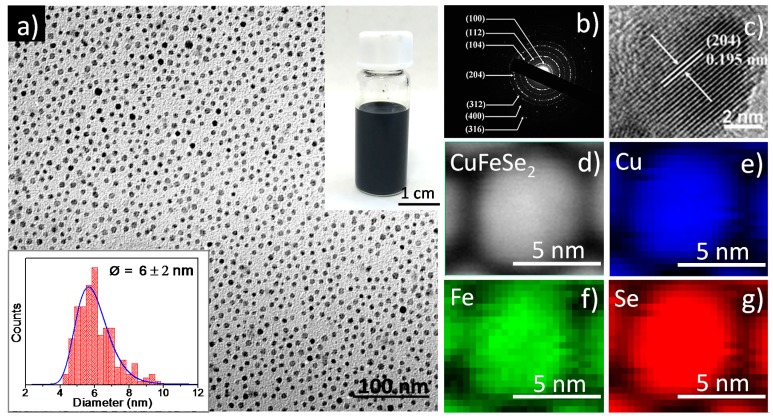
(**a**) general overview transmission electron microscope (TEM) image of the obtained samples, the inset shows the histogram for the measured particle size distribution (6 ± 2 nm) and a photograph of a vial of chloroform solution containing stably-suspended samples; (**b**) selective-area electron diffraction (SAED); (**c**) a general overview high resolution transmission electron microscope (HRTEM) image of the obtained samples; (**d**) annular dark field scanning TEM (ADF-STEM) image of the obtained samples and (**e**–**g**) areal density of each of the elements extracted from the electron energy loss spectroscopy (EELS) spectrum image.

**Figure 3:**
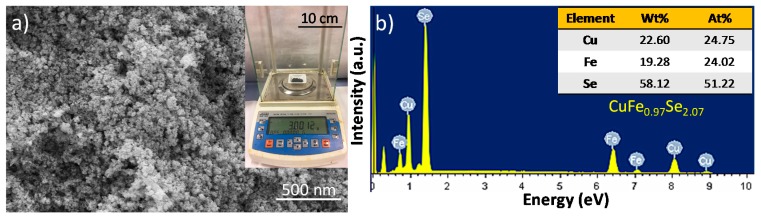
(**a**) The scanning electron microscope (SEM) image shows the surface-clean samples after ligand removal, and the inset in (**a**) shows the typical yield of the samples in this work by one large-scale synthesis (~3 g); (**b**) energy dispersive X-ray spectrometer (EDX) spectrum of the obtained samples and quantitative analysis of the as-synthesized samples with a formula of CuFe_0.97_Se_2.07_ in accordance to the atomic number ratio.

Electron microscopy was employed to characterize the morphology of the product. Figure 2a shows a transmission electron microscope (TEM) image of the as-prepared CuFeSe_2_ NCs, highlighting the uniformity and quasi-spherical morphology of the product. The particle size distribution is shown in the inset of Figure 2a, and the average diameter of NCs was 6 ± 2 nm. The product can be well dispersed in non-polar solvents before ligand exchange, such as toluene or chloroform, forming a stable, dark dispersion (inset of Figure 2a). The seven diffraction rings of the obtained samples corresponding to the (100), (112), (104), (204), (312), (400) and (316) planes can be clearly seen from the selected area electron diffraction pattern (Figure 2b). Further structural characterization was done by HRTEM (Figure 2c). The lattice spacing was measured to be 1.95 Å, corresponding to the expected lattice spacing for the (204) plane of CuFeSe_2_ (Figure 2c). Figure 2d–g shows the results from EELS analysis performed in the sample. The elemental mapping in a region containing the particles is shown and reveals that all elements expected (Cu, Fe and Se) are present in the sample and are equally distributed throughout all the particles. Furthermore, this synthesis protocol was optimized to obtain more than 3.0 g of NCs per batch with a 95% material yield (Figure 3a), which was the amount required for a complete characterization of the material at the laboratory scale. The SEM-EDX shows the stoichiometric ratio of CuFe_0.97_Se_2.07_ (Figure 3b), which is very close to the stoichiometric ratio of 1:1:2. 

**Figure 4:**
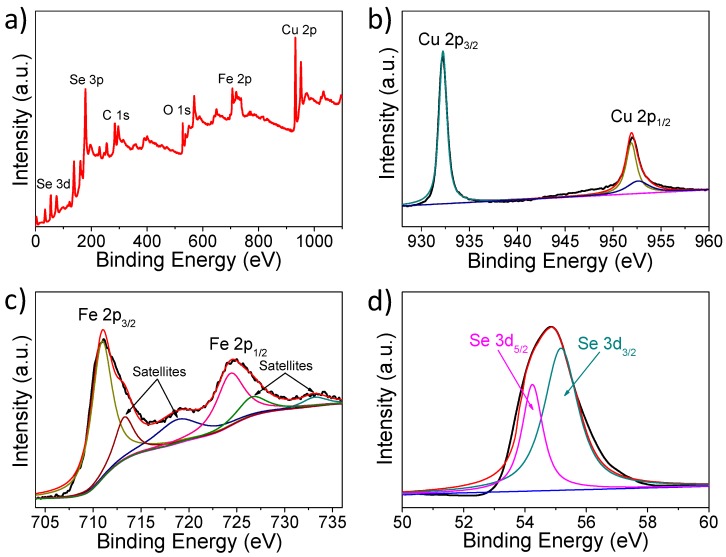
The X-ray photoelectron spectroscopy (XPS) spectra of the obtained samples: (**a**) survey spectrum of surface-cleanCuFeSe_2_; (**b**) Cu 2p; (**c**) Fe 2p; (**d**) Se 3d.

To further confirm the valence states of the individual elements in the obtained samples, the surface-clean products are characterized by XPS. The XPS spectra in the range of 0~1100 eV are shown in Figure 4a. The X-ray photoelectron peaks of Cu (2p), Fe (2p), Se (3d) C 1s (284.6 eV) and O 1s (531.5 eV) are displayed. Two characteristic Cu 2p peaks are located at 932.1 eV (2p_3/2_) and 951.9 eV (2p_1/2_) with a binding energy splitting of 19.8 eV, indicating the presence of Cu^1+^ (Figure 4b) [39]. The 2+ valence 2p_3/2_ and 2p_1/2_ peaks are located at 933.7 eV and 953.6 eV, respectively. There is a weak peak (at 953.6 eV) much closer to the Cu^2+^2p_1/2_ reported [40], which reveals that the valences of Cu are mainly +1 and probably contain a very small contribution of +2. The binding energies are at 711 eV (2p_3/2_) and 724.6 eV (2p_1/2_) with the peak energy difference of 13.6 eV, which is in agreement with the reported Fe^3+^ spectrum (Figure 4c) [41,42]. Normally, the 2p peaks have associated satellite peaks that may partially overlap the main peaks. According to what has been reported, the satellite peak of Fe 2p_3/2_ for the oxidation states is located ~8 eV higher than the main Fe 2p_3/2_ peak [42,43]. Thus, the main satellite peak obtained at ~719 eV can be seen clearly in this work. In addition, there are some other weak satellite peaks for Fe 2p_1/2_ at 729 eV and 733 eV. From Figure 4d, the Se 3d_5/2_ and 3d_5/2_ peaks are confirmed at 54.3 eV and 55.1 eV, which is consistent with the values reported previously and can be assigned to Se^2−^ [44,45,46].

**Figure 5:**
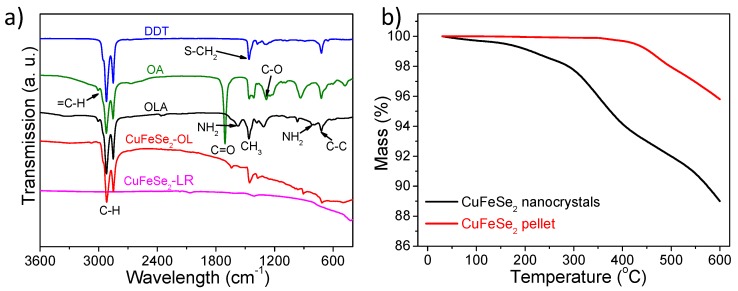
(**a**) Fourier transform infrared spectroscopy (FTIR) spectra of dried CuFeSe_2_ before (CuFeSe_2_-organic ligands; CuFeSe_2_-OL) and after (CuFeSe_2_-ligands removal; CuFeSe_2_-LR) ligands’ removal; FTIR spectra of pure solvent dodecanethiol (DDT), oleic acid (OA) and oleylamine (OLA) are shown as well, respectively; (**b**) thermal gravimetric analyses (TGA) of the CuFeSe_2_, black and red curves refer to the results of the samples before (original NCs) and after pellet fabrication, respectively.

The organic groups on the surface of the product hinder the transfer of electrons among the nanomaterials and reduce the conductivity of the product. To promote the charge transport of samples, the organic groups in the surface of CuFeSe_2_ NCs should be thoroughly removed. Among the potential candidates to remove organic ligands from the CuFeSe_2_ NCs surface, we employed hydrazine, although it is toxic and dangerous to manipulate, because it is very efficient. Figure 5a shows the FTIR spectra of the products before and after removal of organic groups. From the figure, we can see that the original sample spectra show a strong absorption peak in the high frequency region (2850–3000 cm^−1^) and various absorption peaks in the low-frequency region (700–1650 cm^−1^). However, these peaks disappeared after the sample had undergone washing treatment by a mixed solution of hydrazine hydrate and hexane, indicating that the organic groups on the NCs’ surface were removed completely. In addition, using other non-toxic inorganic salts can be considered for the ligand removal in order to implement this material in TE devices in future work, such as sodium salts [47] and ammonium salts [18].

**Figure 6:**
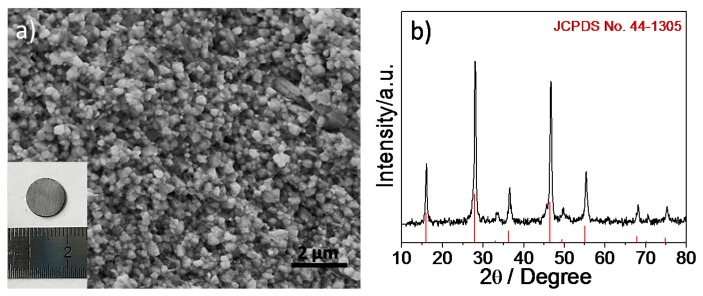
(**a**) SEM image showing the obtained CuFeSe_2_ NCs after the hot-pressing temperature of 623 K; the inset shows a photograph of the hot-pressed pellet; (**b**) XRD pattern of the bulk CuFeSe_2_ nanomaterial pellet.

CuFeSe_2_NCs’ growth was controlled by capping agents. After NCs’ purification by multiple precipitation and redispersion steps, a significant amount of ligands remained attached to the surface. According to TGA (Figure 5b), it allowed us to quantify the amount of the surface ligands at ~7% of the total mass. Meanwhile, the mass loss of the surface clean sample after pellet fabrication did not show an obvious change when the temperature was up to 450 °C, indicating that the pellet has good stability due to the organic ligands’ removal and the hot press process treatment. Some selenium of the pellet probably was lost at the high temperature. As can be seen from Figure 6a, SEM characterization showed large grains after hot pressing at high temperature and high pressure, up to several tens or hundreds of nanometers. The density of the pellet is 5.02 g/cm^3^ using Archimedes’ method, which is approximately 91.8% of the relative theory value. The XRD pattern (Figure 6b) after hot pressing shows that the peak width at half the height of the diffraction peaks became sharp, and the intensity increased significantly, without impurity peaks appearing, indicating that the phase of the sample remained unchanged after hot pressing. The grain size of the bulk nanomaterials further increases to ~120 nm, which was calculated by Scherrer’s equation from the fitting of the XRD pattern.

**Figure 7:**
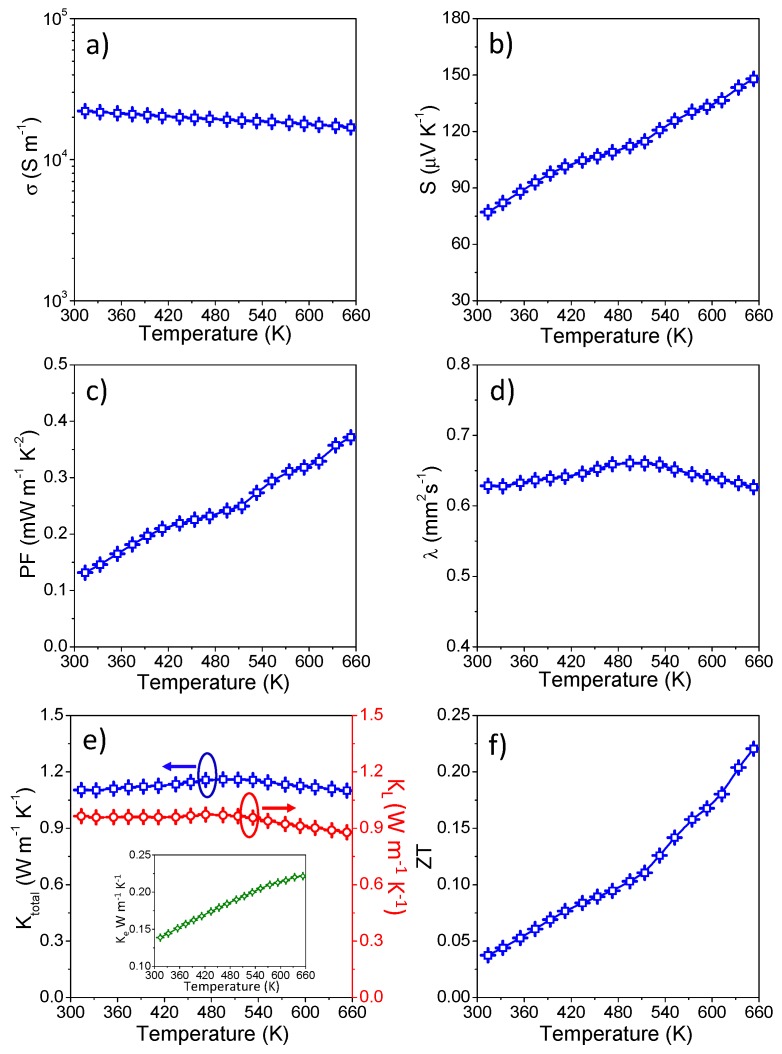
Temperature dependence of (**a**) electrical conductivity (*σ*); (**b**) Seebeck coefficient (*S*); (**c**) power factor (*PF*); (**d**) thermal diffusivity (*λ*); (**e**) thermal conductivity (*κ*_total_) and lattice thermal conductivity (*κ*_L_); the inset shows electronic contribution thermal conductivity (*κ*_e_); (**f**) the figure of merit (*ZT*) of CuFeSe_2_.

The TE transmission properties of the hot-pressed pellet have been measured in the temperature range from room temperature to 653 K, and the results are shown in Figure 7. The electrical conductivity (*σ*) decreases as the temperature increases (Figure 7a), due to lattice defects in the product after hot pressing or carrier scattering from the particle boundary at high temperature caused by the material metalloid [19,21]. The Seebeck coefficient (*S*), shown in Figure 7b, is positive over the entire temperature range, indicating the behavior of the *p*-type semiconductor material, and the majority carriers are holes. It increases with rising temperature, allowing the product to have a higher power factor at high temperatures. The electrical conductivity (*σ*) and the Seebeck coefficient (*S*) were used to calculate the power factor (*PF*) by the formula *PF* = *σ × S*^2^, which in all cases monotonically increases with temperature, and it can be found that the maximum power factor (*PF*) reached ~0.37 mW·m^−1^·K^−2^ at 653 K. 

The thermal diffusivity *λ*, total thermal conductivity (*κ*_total_), lattice thermal conductivity (*κ*_L_) and electron contribution thermal conductivity (*κ*_e_) of the hot-pressed pellet are shown in Figure 7d–e, respectively. From Figure 7d, it can be seen that thermal diffusivity (*λ*) increased slightly before 495 K and then decreased with the temperature increasing. Therefore, similarly, the total thermal conductivity (*κ*_total_ = *λ × C*_p_ *×* *ρ*) of our pristine CuFeSe_2_ nanomaterials increased first and then decreased with increasing temperature, with a maximum value of 1.16 W·m^−1^·K^−1^ at 495 K, as well, which is much lower than that reported of bulk CuFeS_2(1−x)_Se_2x_ samples [48], due to efficient multi-level phonon scattering at point defects, nanomaterials’ grain boundaries and the highly disordered lattice in our bulk nanocrystalline samples as compared to other CuFeX_2_ (X = S, Se)-based bulk materials by solid-state synthesis. The lattice thermal conductivity (*κ*_L_ = *κ*_total_ − *LσT*; here, *L* represents the Lorentz number of 2.0 × 10^−8^ W·Ω·K^−2^) of this pristine CuFeSe_2_ can be calculated with a maximum value of 0.97 W·m^−1^·K^−1^ at 473 K. As illuminated in the inset of Figure 7e, the maximum contribution of electronic thermal conductivity (*κ*_e_ = *κ*_total_ − *κ*_L_) is around 0.22 W·m^−1^·K^−1^ at 653 K in the measured temperature range.

**Figure 8:**
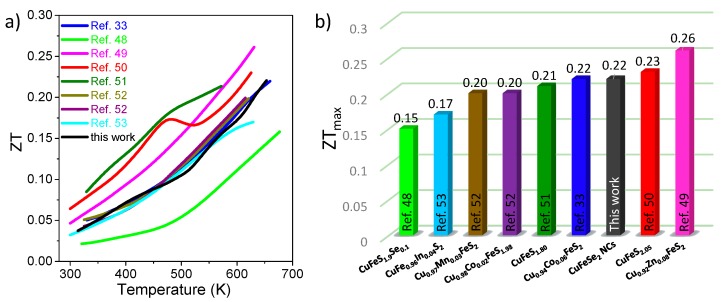
(**a**,**b**) The comparison of our obtained *ZT* value with the reported values by the solid-state technologies for elementally-doped and composition-controlled CuFeX_2_ (X = S, Se) materials.

**Figure 9:**
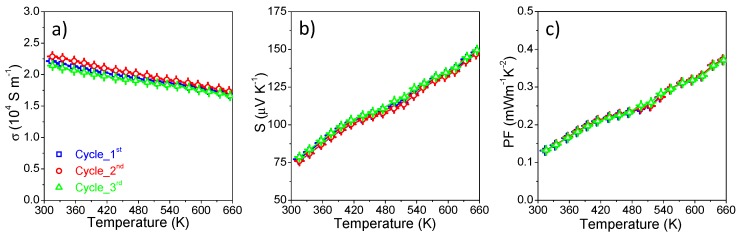
(**a**–**c**) Temperature dependence of the electrical conductivity (*σ*), Seebeck coefficient (*S*) and power factor (*PF*) of nanocrystalline CuFeSe_2_ measured three consecutive times during heating up to 653 K.

Figure 7f shows *ZT* as a function of *T*, in which *ZT* reaches up to 0.22 at 653 K for this pristine CuFeSe_2_ nanomaterial, which is among the largest values obtained for a tellurium-free material comparable with doped CuFeX_2_ (X = S, Se) materials in this similar temperature range (Figure 8) [33,48,49,50,51,52,53], with the additional advantage of low-cost associated with solution processing strategies. Most importantly, the mid-temperature *ZT* has been significantly increased caused by the higher power factor (*PF*) and simultaneously reduced thermal conductivity (*κ*_total_) over a wide temperature range. Furthermore, the *ZT* value can be improved by doping proper elements such as In/Ge/Sb/Sn/S or by controlling the composition, which will be beneficial for our further study of the TE properties of CuFeSe_2_ NCs. The nanocrystalline CuFeSe_2_ materials presented here showed good stability in this work, and the sample was measured three consecutive times during heating up to around 653 K under the same conditions; minor differences were observed (Figure 9).

## 4. Conclusions

In summary, we have developed a novel solution-based strategy for successful large-scale synthesis of uniform and monodisperse CuFeSe_2_ NCs, with high production yields. Furthermore, after ligand removal, the as-prepared NCs were sintered into a high density pellet at the temperature of 623 K for TE application measurements in this work. The largest *ZT* reached up to 0.22 at 653 K for this pristine nanomaterial, which is among the best *ZT* value obtained with a Te-free material in the I-III-VI_2_ group in a similar temperature range, with the additional advantage of abundant components, being environmentally-friendly and low-cost, associated with the solution processing technologies. Taking advantage of this process ability and on the practicality side for the device, this nanomaterial makes TE energy conversion applications possible.
